# Peripheral-blood b-cell subset disturbances in inflammatory joint diseases induced by *Tropheryma whipplei*

**DOI:** 10.1371/journal.pone.0211536

**Published:** 2019-02-27

**Authors:** Maëlle Le Goff, Divi Cornec, Dewi Guellec, Thierry Marhadour, Valérie Devauchelle-Pensec, Sandrine Jousse-Joulin, Marion Herbette, Jean Michel Cauvin, Clara Le Guillou, Yves Renaudineau, Christophe Jamin, Jacques Olivier Pers, Alain Saraux

**Affiliations:** 1 Rheumatology Unit, Centre National de Référence des Maladies Auto-Immunes Rares (CERAINO), Victor Hugo Network, Brest Teaching Hospital, Brest, France; 2 UMR1227, Lymphocytes B et Autoimmunité, Brest University, Inserm, Brest Teaching Hospital, LabEx IGO, Brest, France; 3 DIM and CDC, La Cavale Blanche Hospital, Brest, France; Institut Cochin, FRANCE

## Abstract

**Objective:**

To look for abnormalities in circulating B-cell subsets in patients with rheumatic symptoms of Whipple’s disease (WD).

**Method:**

Consecutive patients seen between 2010 and 2016 for suspected inflammatory joint disease were identified retrospectively. Results of standardized immunological and serological tests and of peripheral-blood B-cell and T-cell subset analysis by flow cytometry were collected. Patients with criteria suggesting WD underwent PCR testing for *Tropheryma whipplei*, and those with diagnosis of WD (cases) were compared to those without diagnosis (controls). We used ROC curve analysis to evaluate the diagnostic value of flow cytometry findings for WD.

**Results:**

Among 2917 patients seen for suspected inflammatory joint disease, 121 had suspected WD, including 9 (9/121, 7.4%) confirmed WD. Proportions of T cells and NK cells were similar between suspected and confirmed WD, whereas cases had a lower proportion of circulating memory B cells (IgD^-^CD38^low^, 18.0%±9.7% vs. 26.0%±14.2%, *P* = 0.041) and higher ratio of activated B cells over memory B cells (4.4±2.0 vs. 2.9±2.2, *P* = 0.023). Among peripheral-blood B-cells, the proportion of IgD+CD27- naive B cells was higher (66.2%±18.2% vs. 54.6%±18.4%, *P* = 0.047) and that of IgD-CD27+ switched memory B cells lower (13.3%±5.7% vs. 21.4%±11.9%, *P* = 0.023), in cases vs. controls. The criterion with the best diagnostic performance was a proportion of IgD+CD27- naive B cells above 70.5%, which had 73% sensitivity and 80% specificity.

**Conclusion:**

Our study provides data on peripheral-blood B-cell disturbances that may have implications for the diagnosis and pathogenetic understanding of WD.

## Introduction

Whipple’s disease (WD) is a rare, systemic, disease caused by the intracellular Gram-positive bacterium *Tropheryma whipplei* (TW). This ubiquitous commensal organism [1] is transmitted among humans via the oro-fecal route [2,3]. WD was first described in 1907. TW was identified by polymerase chain reaction (PCR) in small-bowel biopsies from patients with WD [4–7] in 1991 and later in various samples including stool, saliva, and joint fluid [8, 9]. *T*. *whipplei* is extraordinarily difficult and slow to grow in cultures. The prevalence of TW carriage is highest in adults, residents of rural areas, and exposed individuals such as homeless people and sewer workers [2, 10]. In apparently healthy individuals, the prevalence of carriers identified by PCR screening of stool and saliva was 1.5% to 7.0% and 0.2% to 1.5%, respectively [11–13].

The clinical spectrum of TW infection [14–18] includes classical WD, localized WD [19], acute infection [20], asymptomatic infection, WD influenced by immunosuppression [21], and *T*. *whipplei*-associated arthritis defined as chronic arthritis with a negative duodenal biopsy but positive PCR test at a non-articular site [22]. The non-specific clinical presentation and disease incidence that is too low to warrant routine PCR screening result in major diagnostic challenges. Thus, several years often elapse between symptom onset and the diagnosis [14] of this treatable disease [15]. The reference standard for diagnosing classical WD is duodenal biopsy testing by PCR and periodic acid-Schiff- (PAS) staining [23]. In many of the other forms, the diagnosis relies on PCR testing of saliva, stool, and/or joint fluid, which has a good positive predictive value [8, 11].

Chronic WD and the immune system are closely linked. A contributor to the pathogenesis of WD is the alternatively activated macrophage phenotype, which predominates in the duodenal mucosa and leads to persistent infection by making the macrophages unable to degrade TW [24, 25]. Impaired interleukin (IL)-12 production [26, 27] responsible for decreased IFN-γ production by NK and T cells has been reported in WD [28]. Regulatory T cells are involved in the pathogenesis of WD [29]. Deficiencies in specific peripheral and mucosal T helper cell type 1 (Th1) responses to TW have been reported in patients with WD [30]. The HLA DRB1*13 and DQB1*06 may confer susceptibility to WD [31]. Finally, immunosuppressive therapy may shorten the time from symptom onset to systemic chronic WD, and immunosuppressive therapy in patients with WD may increase the risk of immune reconstitution inflammatory syndrome [32].

To our knowledge, no studies have evaluated the potential role for B cells in WD. Technological advances have improved the phenotypic characterization of blood cells, and flow cytometry is now widely used in patients with hematological, infectious, and systemic auto-immune diseases [33–34]. Abnormalities in the peripheral-blood B-cell subset profile were observed in systemic auto-immune diseases such as primary Sjögren’s syndrome in which the ratio of activated B cells over memory B cells ratio is increased [35] and might serve as a diagnostic aid. We noticed lymphocyte subset abnormalities similar to those seen in primary Sjögren’s syndrome in patients whose symptoms suggested ankylosing spondyloarthritis (inflammatory low back pain) or rheumatoid arthritis (chronic polyarthritis). We then observed the same abnormalities in patients with infectious rheumatic diseases due in particular to *Bartonella* (cat-scratch disease) or TW.

We therefore designed the present study with the aim of describing peripheral-blood lymphocyte subsets, with special attention to B cells, in patients with WD, with rheumatic symptoms. We aimed to assess whether any abnormalities found were sufficiently characteristic to help in diagnosing and monitoring WD.

## Patients and methods

### Participants

We retrospectively collected data on consecutive patients seen at our rheumatology department between April 2010 and December 2016 for suspected inflammatory joint disease. All patients underwent immunological and serological tests, and a peripheral-blood flow cytometry assessment of lymphocyte subsets (total T cells, NK cells, and CD19^+^ B cells) and B-cell subsets (CD19^+^IgD^+^CD38^hi^, transitional, CD19^+^IgD^+^CD27^-^, naive, CD19+IgD+CD27+, unswitched memory, and CD19+IgD-CD27+ switched memory B cells).

### Ethics statement

This study was approved by the CPP Ouest IV ethics committee (2017. CE19). According to the ethics committee recommendations, all data were fully anonymized for analysis and rheumatologists signed a written document which confirmed that all patients received information and were not opposed to the use of their data for this study (non opposition form).

### Identifications of patients with suspected (controls) and confirmed (cases) Whipple’s disease

Within the population, we identified the subgroup of patients (n = 121) who underwent PCR, systematically in stool and saliva, and depending of the symptoms in joint fluid, blood, duodenum, Cerebro Spinal Fluid (CSF), testing for TW. Within this subgroup, we compared the patients with definite diagnosis (cases) vs. no diagnosis (controls) of WD. All cases had at least one clinical criterion suggesting WD, at least one positive PCR test for TW, an antibiotic therapy response recorded by the physician as dramatic and including normalization of C reactive protein and a confirmation of the diagnosis based on all data (exclusion of differential diagnosis) and more than one year of follow up by an independent group of physicians. The cases were divided into three groups depending on whether they had classical WD, focal WD, or chronic TW-associated arthritis (CTWA). Classical WD was defined as a duodenal biopsy positive by PAS staining or TW immunohistochemistry, or as both stool and saliva positive by PCR plus a positive skin biopsy, or as blood positive by PCR. Focal WD was defined as joint fluid positive by PCR but duodenal biopsy negative by PAS staining and immunohistochemistry. CTWA was chronic arthritis plus duodenal biopsy, stool, or saliva positive by PCR but duodenal biopsy negative by PAS staining or immunohistochemistry and joint fluid negative by PCR (non-classical WD) [22].

### Lymphocyte subset analyses

**Fig 1:**
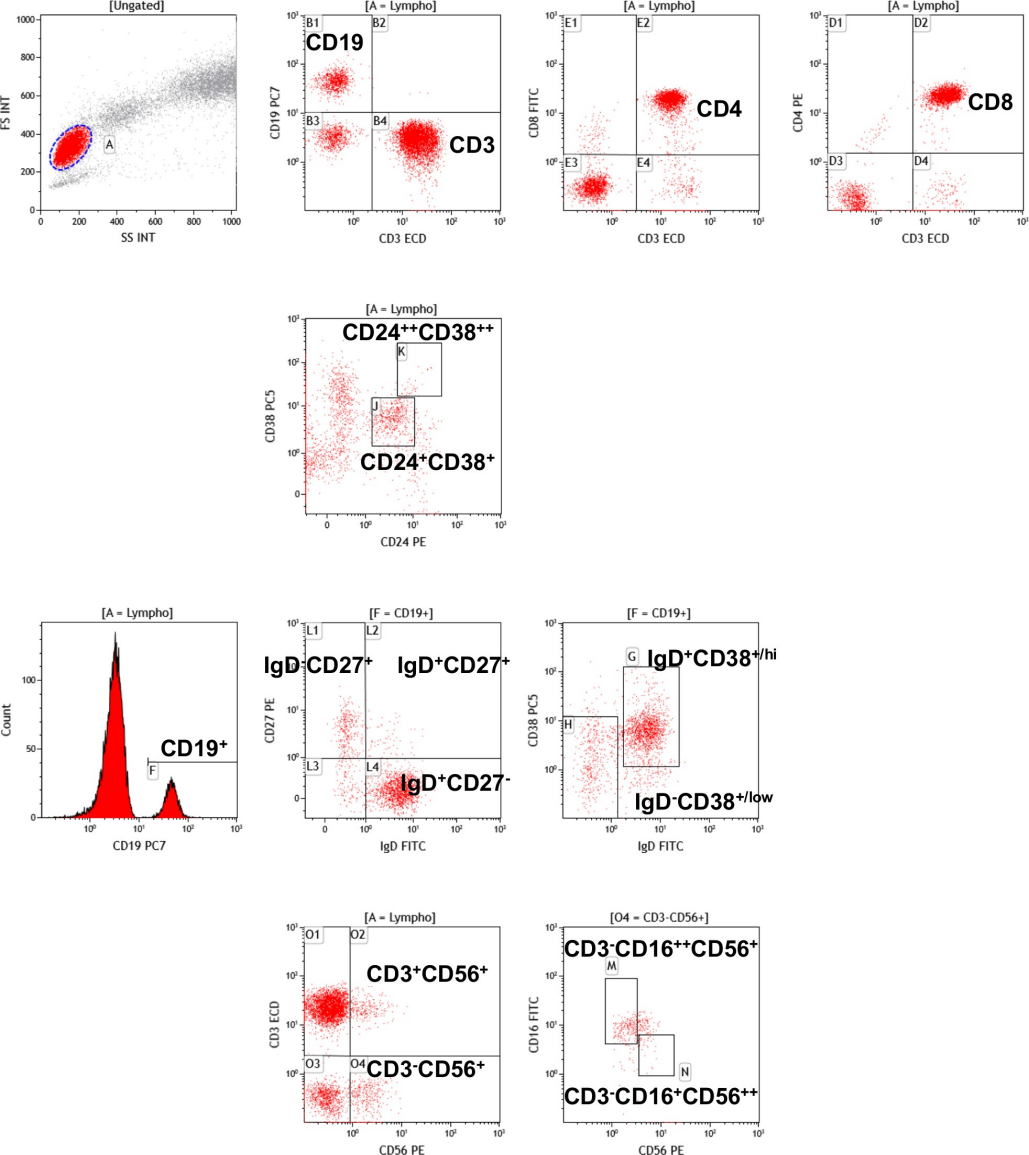
Gating strategy used for fluorescence-activated cell-sorter (FACS). The % of CD3 (CD3^+^CD19^-^), the % of CD4 (CD3^+^CD4^+^), the % of CD8 (CD3^+^CD8^+^) and the % of CD19 (CD3-CD19+) subsets were determined within the total lymphocyte population (gate A = Lympho). For the B cell subsets, the % of CD24^++^CD38^++^ transitional B cells and the % of CD24^+^CD38^+^ naïve B cells were determined within the total lymphocyte population (gate A = Lympho). The % of IgD^+^CD27^-^ naïve B cells, the % of IgD^+^CD27^+^ unswitched memory B cells and the % of IgD^-^CD27^+^ switched memory B cells, and the % of IgD^+^CD38^+/hi^ activated B cells and the % of IgD^-^CD38^+/low^ memory B cells were determined within the B cell subset (gate F = CD19^+^). For the NK cell subsets, the % of CD3^-^CD56^+^ NK lymphocytes and the % CD3^+^CD56^+^ NK-like lymphocytes were determined within the total lymphocyte population (gate A = Lympho). The % of CD3^-^CD16^++^CD56^+^ naïve cytotoxic NK lymphocytes and the % CD3^-^CD16^+^CD56^++^ active NK lymphocytes were determined within the NK lymphocyte population (gate O4 = CD3^+^CD56^+^).

Flow cytometry was used to assess the distributions of CD4+ and CD8+ T cells, NK cells, and total CD19+ B cells(33). All antibodies were purchased from Beckman-Coulter (Hialeah, FL). Phycoerythrin (PE)-cyanine 7 (PC7)-conjugated anti-CD19 monoclonal antibody (mAb) (J4;119) was used to tag B cells; and fluorescein isothiocyanate-conjugated anti-IgD (IA6-2), PE-conjugated anti-CD27 (LS198), and PC5-conjugated anti-CD38 (LS198) mAbs to distinguish among B-cell subsets [36]. In a second B-cell panel, anti-CD19 and anti-CD38 mAbs were combined with PE-conjugated anti-CD24 (ALB9) mAb to identify CD19^+^CD38^hi^CD24^hi^ transitional and CD19^+^CD24^+^CD38^+^ mature B cells. The cells were categorized on an Epics XL (Beckman-Coulter) fluorescence-activated cell-sorter (FACS). All details are summarized in the **Fig 1** and Figure A in S1 File.

### PCR tests and biopsy

The 121 patients with suspected WD had 214 visits and underwent at least one PCR test for TW on a variety of samples (stool; saliva; joint fluid; blood; cerebrospinal fluid; urine; and/or biopsies of lung, skin, and/or duodenal mucosa). Real-time quantitative PCR (qPCR) tests for repeated TW sequences were performed using specific oligonucleotide TaqMan probes(11) at the bacteriology laboratory of the Marseille teaching hospital [37]. Sequencing was performed when an amplified product was detected, followed by a confirmatory PCR test targeting a different TW sequence. Positive and negative controls were used routinely, and the quality of extracted DNA was checked by human actin gene detection [38].

### Statistical analysis

The data were analyzed using the Statistical Package for the Social Sciences (SPSS 25.0, Chicago, IL). Absolute values were described as mean±SD (of number of cells by mm3 or percentages) of circulating lymphocyte subsets. Associations between lymphocyte subset distributions and WD were assessed by univariate analysis using Mann-Whitney test (comparison of continuous data) or Wilcoxon test (comparison of continuous data before and after treatment). Logistic regression was performed to identify the subset most strongly associated with WD. Subset distribution changes over time were then evaluated. *p* values smaller than 0.05 were considered significant. Receiver operating characteristic (ROC) curves were plotted for B-cell subsets at any time point to identify the cutoff associated with the best compromise between sensitivity and specificity.

## Results

### Patient population

Between April 2010 and December 2016, 2917 patients had 3515 visits to our rheumatology department for symptoms suggesting inflammatory rheumatism. Among them, 121 with suspected WD underwent 214 PCR tests for TW. There were 62 (51.2%) men and 59 (48.8%) women with a mean age of 52.5±15 years (range, 16–84 years). Of these 121 patients, 9 had positive PCR tests for TW and were diagnosed with WD: 1 (11.1%) had classical WD, 4 (44.4%) focal WD, and 4 (44.4%) non-classical WD. Of the 112 other patients, 58 received diagnoses of rheumatoid arthritis (n = 24), spondyloarthritis (n = 17), connective tissue disease (n = 4), vasculitis (n = 6), sarcoidosis (n = 3), or other diseases (Lyme disease, sarcoma, or polymyalgia rheumatica, n = 4) and 54 had no diagnosis.

### Detailed features of the 9 patients diagnosed with Whipple’s disease (WD) (Figure B in S1 File)

There were 7 (77.8%) males and 2 (22.2%) females with a mean age of 60.3±11.4 years and a mean symptom duration of 8.5±7 years. Among them, 8 had previously received a diagnosis of rheumatoid arthritis (n = 4, 44.4%), leukocytoclastic vasculitis (n = 1, 11.1%), undifferentiated arthritis (n = 1, 11.1%), spondyloarthritis (n = 1, 11.1%), or calcium pyrophosphate dihydrate deposition disease (n = 1, 11.1%). In the patient with no previous diagnosis, time since symptom onset was only 2 years. In 5 (55.5%) patients, there was a history of treatment with synthetic disease-modifying antirheumatic drugs or TNFα antagonists. Mean serum C-reactive protein was 36.5±24.3 mg/L (range, 0.4–82.9 mg/L), 3 patients had anemia, and 5 patients had hypoalbuminemia.

All 9 patients received first-line hydroxychloroquine (400–600 mg/day) and doxycycline (200 mg/day) treatment for WD, which was consistently effective in resolving the clinical and laboratory abnormalities, with a response time of 10 days to 2 months. The doxycycline was switched to intravenous ceftriaxone after 1 month in 1 patient and to trimethoprim-sulfamethoxazole because of an allergic reaction in another.

Of the 9 patients, 2 are still receiving treatment at the time of writing.

### Lymphocyte subsets in the 9 patients with Whipple’s disease and in the controls

**Table 1** details the peripheral-blood lymphocyte subsets in each patient at baseline. Subset distributions were compared between the group of 9 patients with WD disease and the control group of 112 patients seen for suspected inflammatory joint disease and having a suspicion of WD but negative PCR tests for TW (**Table 2**). The proportions of total lymphocytes, CD4^+^ T cells, CD8 ^+^ T cells, and NK cells were not different between cases and controls. The percentage of circulating IgD^-^CD38^-/low^ memory B cells was significantly lower, and the ratio of IgD^+^CD38^+/hi^ activated B cells over IgD^-^CD38^-/low^ memory B cells significantly higher in cases compared with controls (4.4±2.0 vs. 2.9±2.2, *P* = 0.023). Studying CD27 expression showed that the cases had a higher proportion of IgD^+^CD27^-^ naive B cells (66.2%±18.2% vs. 54.6%±18.4%, *P* = 0.047) and, among memory B cells, a lower proportion of IgD^-^CD27^+^ switched memory cells (13.3%±5.7% vs. 21.4%±11.9%, *P* = 0.023) vs. controls.

**Table 1:** Peripheral-blood B-lymphocyte subsets at baseline of the 9 patients diagnosed with Whipple’s disease (WD).

Patients	1 Classical WD	2 Non-classical WD	3 Non-classical WD	4 Non-classical WD	5 Non-classical WD	6 Focal WD	7 Focal WD	8 Focal WD	9 Focal WD
Total lymphocytes	730	2160	1060	1290	930	1530	1980	1160	1730
IgD^-^CD38^-/low^ memory B cells, N/mm^3^ (%)	3 (7.8)	18 (11.2)	16 (22.2)	12 (13.0)	11(12.0)	19 (11.3)	25 (12.0)	14 (26.7)	50 (22.4)
IgD^+^CD27^-^ naive B cells, N/mm^3^ (%)	11 (29.0)	144 (87.5)	46 (66.4)	65 (70.6)	78 (84.5)	147 (87.1)	152 (74.2)	36 (70.8)	137 (60.8)
Ratio of IgD^+^CD38^+/hi^ activated B cells over IgD^-^CD38^-/low^ memory B cells	5.5	7.4	2.8	5.6	6.6	7.1	5.9	2.3	2,6
IgD^-^CD27^+^ switched memory B cells, N/mm^3^ (%)	3 (8.2)	10 (6.2)	11 (15.5)	12 (12.7)	9 (10.1)	9 (5.5)	34 (16.6)	5 (9.8)	41 (18.2)

**Table 2:** Peripheral-blood B-cell subsets (mean±SD of the number by mm^3^ and %) in patients with Whipple’s disease, compared to controls with inflammatory diseases but no Whipple’s disease among patient who had a suspicion of Whipple’s disease (at least one PCR).

Lymphocyte subset	Whipple’s disease 9	No Whipple’s disease 112	*P* value
Total lymphocytes, N/mm^3^	1708±575	2147± 953	0.21
CD3 (%)	73.0±10.0	73.8±8.4	0.96
CD4 (%)	52.1±10.4	50.8±9.0	0.63
CD8 (%)	18.9±6.8	21.6±8.1	0.42
CD4/CD8 ratio	3.4±2.4	2.8±1.7	0.42
CD19 (%)	10.2±5.3	21.7±112.5	0.43
IgD^+^CD38^+/hi^ activated B cells (%)	64.1±14.4	51.9±18.7	0.054
IgD^-^CD38^-/low^ memory B cells (%)	18.0±9.7	26.0±14.2	**0.041**
ratio of IgD^+^CD38^+/hi^ activated B cells over IgD^-^CD38^-/low^ memory B cells	4.4±2	2.9±2.2	**0.023**
IgD^+^CD27^-^ naive B cells (%)	66.2±18.2	54.6±18.4	**0.047**
IgD^+^CD27^+^ unswitched memory B cells (%)	8.5±9.4	12.4±11.6	0.17
IgD^-^CD27^+^ switched memory B cells (%)	13.3±5.7	21.4±11.9	**0.023**
CD24^++^ CD38^++^ transitional B cells (%)	1.0±1.7	1.2±2.1	0.98
CD24^+^ CD38^+^ mature B cells (%)	16.5±16.2	16.4±16.6	0.83
CD3- CD56^+^ NK lymphocytes (%)	8.2±9.9	6.4±5.1	0.98
CD3- CD16^++^CD56^+^ naive cytotoxic NK lymphocytes (%)	47.7±21.7	56.7±20.6	0.22
CD3- CD16^+^ CD56^++^ active NK lymphocytes (%)	10.5±14.6	10.8±12.5	0.93
CD3^+^CD56^+^ NK-like lymphocytes (%)	1.3±1.2	2.0±2.5	0.51

We compared the 22 visits by the 9 patients with WD to the 3493 visits by the 2908 patients without PCR testing for TW or with negative PCR testing for TW (**Table 3**). The differences between the two groups were similar to those found at baseline. In addition, the lymphocyte counts were lower in the patients with WD than without a diagnosis of WD.

**Table 3:** Peripheral-blood B-cell subsets (mean±SD) in patients with Whipple’s disease compared to controls with inflammatory disease but negative PCR tests for Whipple’s disease, or without PCR tests for WD.

B-cell subset	Whipple’s disease 22 visits	No Whipple’s disease 3493 visits	*P* value (Wilcoxon test)
Total lymphocytes, N/mm^3^	1575.9 ±474.10	2071.82±1086.99	0.005
IgD^-^CD38^-/low^ memory B cells (mean of %)	16.8±5.99	24.14±13.22	0.001
Ratio of IgD^+^CD38^+/hi^ activated B cells to IgD^-^CD38^-/low^ memory B cells (mean of %)	4.61±1.85	3.69±4.66	0.001
IgD^+^CD27^-^ naive B cells (mean of %)	72.33±12.97	59.03±18.67	<0.001
IgD^-^CD27^+^ switched memory B cells (mean of %)	12.89±5.01	19.03±11.38	0.006

Some patients with WD were receiving glucocorticoid (3 patients), methotrexate (5 patients) or TNFα antagonist (2 patients) therapy. These treatments may modify B-cell subset distribution. However, in the controls, patients were taking the same treatments (20 received glucocorticoids, 19 methotrexate, and 7 a biologic).

### Changes in lymphocyte subset distribution in the 9 patients during treatment for Whipple’s disease (WD)

Flow cytometry was performed before the diagnosis (i.e., probably after disease onset, as symptoms were present), at the diagnosis of WD, and during treatment in the 9 patients with WD. No changes occurred in lymphocyte subset distribution (**Table 4**). Four patients had an evaluation at the end of the treatment and there was not changes (Figure C in **S1 File**: example on IgD^-^CD27^+^ switched memory B cells) despite the end of treatment by methotrexate (3 patients), TNF inhibitor (1 patient) and steroids (2 patients).

**Table 4:** Comparison of peripheral-blood B-cell subsets (mean of %) before and after starting treatment for Whipple’s disease in 9 patients.

	Before treatment	During treatment	*P* value (Wilcoxon test)
IgD^-^CD38^-/low^memory B cells	20.48±7.56	15.78±5.49	0.31
Ratio of IgD^+^CD38^+/hi^ activated B cells over IgD^-^CD38^-/low^ memory B cells (	4.09±2.49	4.47±1.42	1.00
IgD^+^CD27^-^ naive B cells	72.18±10.27	65.74±17.64	0.87
IgD^-^CD27^+^ switched memory B cells	12.6±8.05	12.90±4.06	1.00

### Relevance of the B-cell subset distribution to the diagnosis of Whipple’s disease (WD)

**Fig 2:**
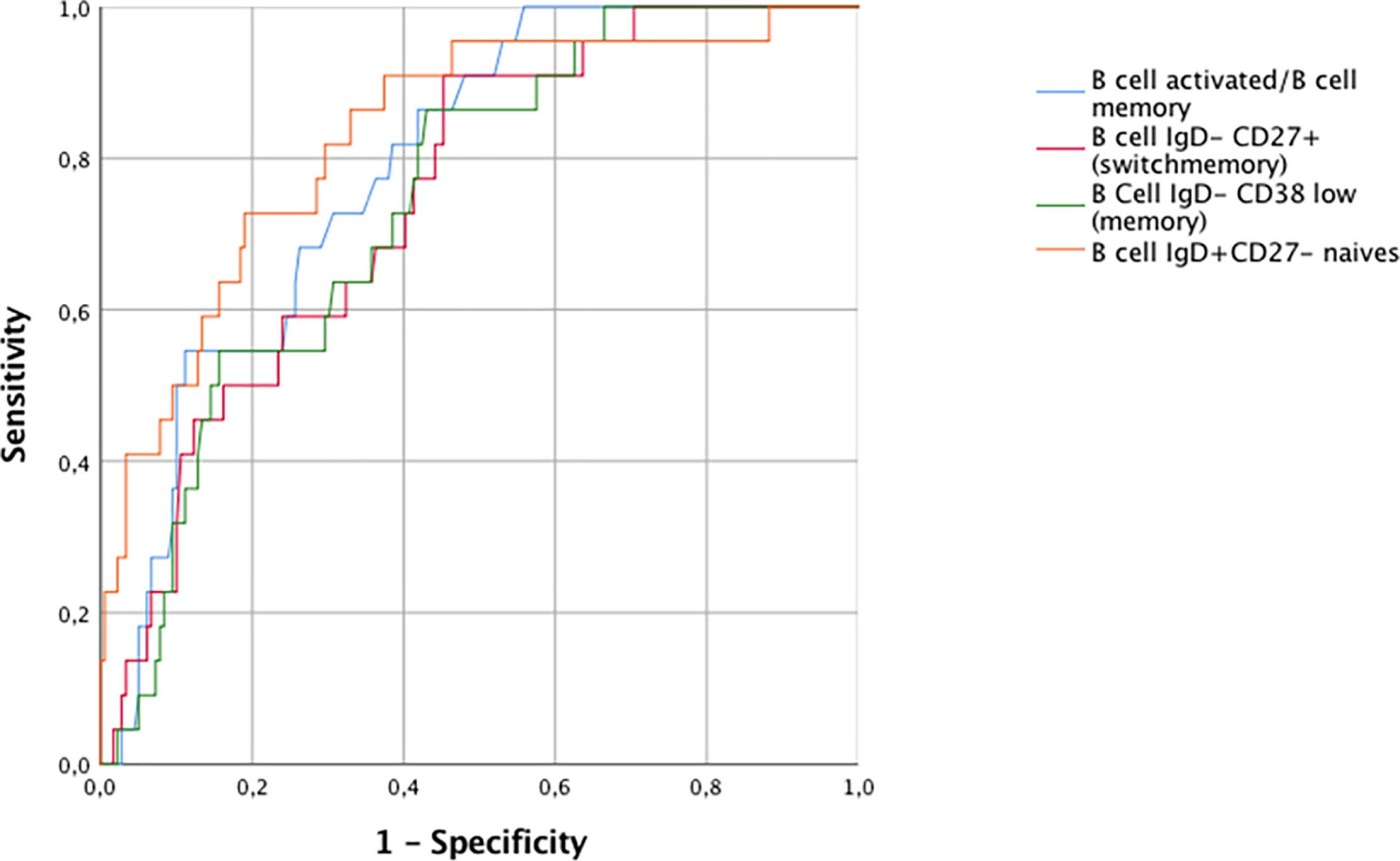
ROC curves of the diagnostic performance of B-cell subset distribution for Whipple’s disease. The best curve was obtained with IgD^+^CD27^-^ naive B cells (%).

Full B-cell distribution was evaluated at 198 visits. ROC curve analysis showed that the proportion of IgD^+^CD27-naive B cells provided the best compromise between sensitivity and specificity for the diagnosis of WD. With a cutoff of 70.5%, the area under the curve was 0.83, sensitivity was 73.0%, and specificity was 80.0% (**Fig 2**). If we limit the controls to patients with autoimmune disease, results were quite similar (the area under the curve was 0.79).

Of 19 visits by patients with WD having all subpopulation evaluations, 14 (73.7%) were associated with a proportion of IgD^+^CD27-naive B cells at or above the cut-off, versus 35 (19.5%) of 179 visits by patients without WD (*P*<0.0001). Interestingly, the proportion of IgD+CD27-naive B cells was not elevated in patients with Lyme disease, reactive arthritis, or septic arthritis.

## Discussion

In patients with WD and rheumatic symptoms, the distribution of peripheral-blood B-cell subsets differed from that in controls with inflammatory joint disease. No differences were found, in contrast, for total lymphocytes, T cells, or NK cells. The cases had lower proportions of circulating IgD^-^CD38^-/low^ memory B cells and, most notably, of IgD-CD27+switched memory B cells. The ratio of IgD^+^CD38^+/hi^ activated B cells over IgD^-^CD38^-/low^ memory B cells was higher in the cases, because of a higher percentage of IgD+CD27- naive B cells. The best diagnostic performance was obtained for an IgD^+^CD27^-^ naive B-cell proportion at or above 70.5%. The B-cell subset abnormalities documented in our study may provide diagnostic assistance, in combination with the medical history, physical findings, and standard laboratory tests. Furthermore, they may help to understand the pathophysiology of WD. In our population, patients with other infectious diseases did not have the B-cell subset abnormalities seen in the patients with WD.

WD is characterized by massive infiltration of TW in the duodenal mucosa, lack of duodenal inflammation, malfunction of antigen-presenting cells, and alternative activation of macrophages [24]. Dysregulation of T-cell functions are involved in the pathogenesis of WD. The proportion of CD4^+^ T cells in peripheral blood and the lamina propria is reduced, and both T-cell activation and the Th1 response are impaired, with diminished production of IL-2 and IFN-gamma [30]. These deficiencies allow the establishment of chronic TW infection in susceptible patients [31]. Regulatory T cells are abundant in the duodenal mucosa and exhibit enhanced activity in peripheral blood, leading to insufficient bacterial clearance [31]. However, a primary T cell defect does not appear to be the cause for chronic WD [29]. Our patients did not exhibit these previously described T-cell abnormalities; more specifically, they had no decrease in the proportion of CD4^+^ T cells [30].

B-cell abnormalities reported previously in WD include serological alterations and changes in duodenal mucosal B cells [39–40]. Thus, WD is associated with the HLA-DRB1*13 and DQB1*06 genotypes [31], which may preferentially present antigenic epitopes to stimulate humoral responses instead of cellular immune responses. More interestingly, it was recently found that a single rare non-synonymous mutation with age-dependent incomplete penetrance leading to *IRF4* deficiency which can underlie WD. As IRF4 help B-cell development, a genetic mechanism may explain why the lymphocyte subsets in our patients remained unchanged (despite antibiotic, infection by TW remains and induces altered kinds of B cells) [41]. Another hypothesis may be that the disease induced irreversible subset distribution abnormalities.

Our study has three main limitations. First, the number of patients was small. Second, at the time of flow cytometry, some patients were receiving or glucocorticoid (4 patients), methotrexate (4 patients) or TNFα antagonist (2 patients) therapy. These treatments are known to modify B-cell subset distribution [42, 43]. However, in the controls, patients were taking the same treatments (20 received glucocorticoids, 19 methotrexate, and 7 a biologic). Third, the controls were not healthy individuals but patients with inflammatory diseases such as Sjogren’s syndrome, which are known to be associated with alterations in peripheral-blood B-cell subset distribution [33, 35, 44].

To conclude, flow cytometry analysis of peripheral-blood lymphocytes in patients with rheumatic symptoms and a diagnosis of WD showed alterations in B-cell subset distribution compared to controls with inflammatory diseases, a clinical suspicion of WD, but negative PCR tests for WD. Treatment for WD consistently induced a clinical response but did not change the abnormalities in B-cell subset distribution. These abnormalities are not sufficiently characteristic to serve as a diagnostic tool when considered alone but may provide guidance when combined with other criteria. The B-cell subset abnormality associated with the best compromise between sensitivity and specificity for diagnosing WD was a proportion of IgD^+^CD27-naive B cells ≥70.5%. Our study provides the first data on peripheral-blood B-cell subset alterations in WD and may suggest hypotheses regarding the role for immunological abnormalities in this condition.

## Supporting information

S1 File Method for fluorescence-activated cell-sorter (FACS) (Figure A). Features of the 9 patients diagnosed with Whipple’s disease (WD) (Figure B). Lack of changes of IgD-CD27+ switched memory B cells in 4 patients who had evaluation before treatment, under treatment and at the end of treatment (Figure C).
(DOCX)
