# Supplementary material for: Peripheral-blood b-cell subset disturbances in inflammatory joint diseases induced by Tropheryma whipplei
Source: PLoS One. 2019 Feb 27;14(2):e0211536. doi: 10.1371/journal.pone.0211536 (PMC6392227; doi:10.1371/journal.pone.0211536)
Supplement: S1 File — Features of the 9 patients diagnosed with Whipple’s disease (WD) (Figure B). Lack of changes of IgD-CD27+ switched memory B cells in 4 patients who had evaluation before treatment, under treatment and at the end of treatment (Figure C). (DOCX) [file pone.0211536.s001.docx]

**Figure A in S1 file :** Method for fluorescence-activated cell-sorter (FACS).

*Different cocktails of fluorochrome-associated antibodies were used:*

| *Cocktail* | *N° 1* | *N° 2* | *N° 3* | *N° 4* |
| --- | --- | --- | --- | --- |
| *Antibodies* | *FITC CD8*  *PE CD4*  *ECD CD3*  *PC7 CD19* | *FITC IgD*  *PE CD27*  *PC5 CD38*  *PC7 CD19* | *PE CD24*  *PC5 CD38*  *PC7 CD19* | *FITC CD16*  *PE CD56*  *ECD CD3* |

*- Quality control:*

*The panels were initially established using isotype controls, single antibody staining and complete antibody cocktails for the determination of the PMT values and the calculation of the compensation matrices.*

*- Information on the material used:*

*All stainings were performed on whole blood and all samples were prepared using the Immunoprep reagent system (Beckman Coulter) for the lysing of the red blood cells and the fixation of the white blood cells, which is a rapid and no-wash standardized procedure for maximizing reproducibility.*

*-Reference range for healthy controls:*

*According to the literature (Marieke Comans-Bitter W et al., J Pediatr, 1997, 130(3):388-93 and Kverneland AH et al, Cytometry Part A, 2016, 89A, 543-564), we use the reference ranges described in the table below from healthy controls for the CD3, CD4, CD8, CD19 and CD56 populations.*

| *Total lymphocytes* | *CD3* | | *CD4* | | *CD8* | | *ratio* | *CD19* | | *CD56* | |
| --- | --- | --- | --- | --- | --- | --- | --- | --- | --- | --- | --- |
| */mm3* | *%* | */mm3* | *%* | */mm3* | *%* | */mm3* |  | *%* | */mm3* | *%* | */mm3* |
| *1500-4000* | *56-90* | *540-2738* | *35-72* | *300-1793* | *9-48* | *95-1157* | *1.5-2.5* | *4-26* | *55-681* | *4-30* | *52-537* |

*- Definition of the cell subpopulations.*

*We believe that the B cell definition based on the IgD/CD27 distribution associated with the definition based on the IgD/CD38 distribution is more informative than the usage of only one. As seen in the figure below, the IgD+CD27- naïve B cells contain the IgD+CD38+/hi activated B cells (green population in part A), but IgD+CD38+/hi activated B cells encompass the IgD+CD27- naïve B cells and the IgD+CD27+ unswitched memory B cells (violet population in part B). The IgD-CD38+/low memory B cells encompass the IgD-CD27+ switched memory B cells and also the IgD-CD27- B cells (green population in part C). For these reasons, we decided to evaluate all the subpopulations independently.*

**Figure B in S1 File.** Features of the 9 patients diagnosed with Whipple’s disease (WD)

| Patient | 1  Classical WD | 2  Non-classical WD | 3  Non-classical WD | 4  Non-classical WD | 5  Non-classical WD | 6  Focal WD | 7  Focal WD | | 8  Focal WD | | 9  Focal WD | |
| --- | --- | --- | --- | --- | --- | --- | --- | --- | --- | --- | --- | --- |
|  | Clinical presentation at diagnosis | | | | | | | | | | | |
| Age (years) | 75 | 62 | 64 | 55 | 42 | 67 | 70 |  | | 65 | 43 | |
| Sex | Male | Male | Male | Female | Male | Male | Male |  | | Male | Female | |
| Symptom duration (years) | 19 | 7 | 4 | 2 | 3 | 11 | 21 |  | | 5 | 5 | |
| First diagnosis considered | Rheumatoid arthritis | Leukocytoclastic vasculitis | Rheumatoid arthritis | - | Spondyloarthritis | Undifferentiated arthritis | Rheumatoid arthritis |  | | CCPD | Rheumatoid arthritis | |
| Rheumatic symptoms | Arthralgia, Arthritis | Arthralgia, Arthritis, inflammatory low back pain | Arthralgia | Arthralgia, Arthritis | Arthralgia, Arthritis | Arthralgia, Arthritis | Arthralgia, Arthritis |  | | Arthralgia, Arthritis | Arthralgia, Arthritis inflammatory low back pain | |
| Weight loss | + | - | + | - | - | + | - |  | | - | - | |
| Other | Fever | Abdominal pain Lymphadenopathy Pruritus, episcleritis | Pericarditis | Pericarditis | Fever, Pericarditis |  | Sicca,  proteinuria |  | |  | Diarrhea, Abdominal Pain | |
| Treatment at M0 | no | Methotrexate  TNF inhibitor  Gluco  corticoids | Methotrexate | no | Methotrexate  TNF inhibitor | Methotrexate | no |  | | Methotrexate  Gluco  corticoids | Gluco  corticoids | |
|  | Treatment | | | | | | | | | | | |
| Treatment | DX + HQN  2 Weeks CTX | DX + HQN | DX + HQN | DX + HQN | DX + HQN | DX + HQN  Then  TMP-SMX | DX + HQN |  | | DX + HQN  Then  TMP-SMX | DX + HQN | |
| Treatment duration (months) | 48 months | 44 months | 18 months | 30 months | 18 months | 24 months | Ongoing  (24 months) |  | | Ongoing  (20 months) | 24 months | |
|  | Diagnosis | | | | | | | | | | | |
| Tests for Whipple’s disease | PCR on stool, saliva, urine, blood, duodenum  Duodenal biopsy: PAS staining, PCR | PCR on stool, saliva | PCR on stool, saliva duodenum  Duodenal biopsy: PAS staining, PCR | PCR on stool, saliva | PCR on stool, saliva, blood, duodenum  Duodenal biopsy: PAS staining, PCR | PCR on stool, saliva, joint fluid, blood, duodenum, CSF  Duodenal biopsy: PAS staining, PCR | PCR on stool, saliva, joint fluid |  | | PCR on stool, saliva, joint fluid, duodenum  Duodenal biopsy: PAS staining, PCR | PCR on stool, saliva, joint fluid, duodenum  Duodenal biopsy: PAS staining,  PCR | |
| Positivity | PCR: Stool, saliva  Duodenal biopsy by PAS staining | PCR: Stool, saliva | PCR: Stool, saliva | PCR: Stool | PCR: Stool | PCR: Stool, saliva, joint fluid | PCR: Stool, joint fluid |  | | PCR: Stool, saliva, joint fluid | PCR: Stool, joint fluid | |
|  | Biological and radiological presentation at diagnosis | | | | | | | | | | | |
| CRP (mg/L) | 82.9 | 40.8 | 38.8 | 31 | 15.7 | 11. 5 | 51 |  | | 34 | | 0.4 |
| Anemia | + | + | + |  |  |  |  |  | |  | |  |
| Hypoalbuminemia | + | + | + |  |  | + |  |  | | + | |  |
| Radiologic erosions | + |  |  | + |  |  |  |  | |  | |  |

CSF: Cerebrospinal Fluid; PAS: Periodic Acid Schiff; CRP: C-Reactive Protein; DX: Doxycycline (200 mg/day); HQN: Hydroxychloroquine (400-600 mg/day); CTX: Ceftriaxone; TMP-SMX: trimethoprim/sulfamethoxazole (160/800 mg twice daily)

**Figure C in S1 File :** Lack of changes of IgD-CD27+ switched memory B cells in 4 patients who had evaluation before treatment, under treatment and at the end of treatment.

| IgD+CD27- naive B cells | End of ttt | During treatment | Before treatment |
| --- | --- | --- | --- |
| Patient 2 | 81.3 | 85.7 | 84.5 |
| Patient 3 | 85.7 | 81.5 | 87.5 |
| Patient 7 | 83.8 | 82.6 | 87.1 |
| Patient 8 | 82.3 | 75.8 | 70.8 |
